# Global, regional, and national trends in pulmonary arterial hypertension burden, 1990–2021: findings from the global burden of disease study 2021

**DOI:** 10.3389/fpubh.2025.1516365

**Published:** 2025-05-29

**Authors:** Zhenhao Liu, Liumei Mo, Wenjing Cao, Kui Wang, Hanxian Gong, Chen Li, Wei Pan, Jinqing He

**Affiliations:** ^1^Department of Cardiovascular Medicine, Pingxiang People's Hospital, Jiangxi, China; ^2^Department of Cardiology, Foshan Women and Children Hospital, Foshan, China; ^3^Department of Geriatrics, Foshan Women and Children Hospital, Foshan, China; ^4^The First Clinical Medical College, Qilu Hospital of Shandong University, Jinan, China; ^5^Department of Cardiology, Ganzhou People's Hospital, Ganzhou, China

**Keywords:** pulmonary arterial hypertension, disease burden, disability-adjusted life years, incidence, death

## Abstract

**Objectives:**

Pulmonary arterial hypertension (PAH) is a severe and life-threatening condition. This study systematically examines the global epidemiology of PAH, focusing on trends in incidence, mortality, and disability-adjusted life years (DALYs) over the past 32 years to inform evidence-based policy and healthcare strategies.

**Methods:**

Data from the Global Burden of Disease (GBD) 2021 study was used to analyze PAH incidence, mortality, and DALYs globally, regionally, and nationally from 1990 to 2021. Age-standardized incidence rate (ASIR), death rate (ASMR), disability-adjusted life years rate (ASDR) and estimated annual percentage change (EAPC) were assessed by age, gender, and socio-demographic index (SDI) quintiles. Hierarchical cluster analysis was performed to evaluate the temporal patterns of disease burden changes across GBD regions.

**Results:**

Global PAH incident cases increased by 85.6%, from 23,301 in 1990 to 43,251 in 2021. ASIR increased slightly from 0.50 to 0.52 per 100,000 persons (EAPC 0.05%). From 1990 to 2021, PAH-related deaths increased from 14,842 to 22,021, though ASMR decreased (EAPC -0.57%). In 2021, PAH accounted for 642,104 DALYs, with ASDR showing a downward trend (EAPC -1.31%). Regions with low SDI exhibited the highest ASIR, while both ASMR and DALYs decreased across all SDI categories. Southern Sub-Saharan Africa had the highest incidence, while Central Asia saw the largest increases in mortality and DALYs.

**Conclusion:**

Over the past 32 years, global ASMR and ASDR for PAH have decreased, while ASIR showed a modest increase. Persistent imbalances in treatment and outcomes remain in certain regions. Enhanced prevention and comprehensive management strategies are needed to diminish the global PAH burden and improve health equity.

## Introduction

Pulmonary arterial hypertension (PAH) is a severe disorder characterized by elevated pulmonary artery pressure resulting from increased pulmonary vascular resistance (1). Without appropriate treatment, PAH typically progresses to right ventricular failure and ultimately leads to mortality, contributing to high morbidity and mortality rates (2). The global disease burden of PAH is rapidly evolving due to advances in medical diagnostics and treatments, accelerated ageing of the global population, and the multifaceted influence of socio-economic factors such as dietary habits and exposures to occupational and environmental toxins. Since its initial characterization, the demographic profile of PAH patients has shifted, with younger women of childbearing age now recognized as the prototypical individuals with idiopathic PAH. Changes in estrogen levels and its metabolic pathways are conncened with an increased risk of PAH in females, highlighting the hormone’s modulatory role in disease susceptibility (3, 4).

Despite extensive research, the precise pathophysiologic mechanisms of PAH remain unclear (5–7). PAH encompasses a spectrum of pathophysiological phenotypes, including pulmonary artery smooth muscle cell (PASMC) hyperproliferation, pulmonary artery endothelial cell (PAEC) dysfunction, metabolic reprogramming (such as the Warburg effect), impaired angiogenesis, resistance to apoptotic resistance, chronic inflammation, and phenotypic plasticity (8, 9). The current clinical guidelines are based on these pathophysiological mechanisms and recommend combination therapies (10, 11, 35, 36). Despite notable advancements in diagnostic and therapeutic modalities in recent years, there remains a critical need for a deeper comprehension of the global disease burden of PAH to better understand its specific impact on public health. Which continues to represent a significant global health issue, necessitating ongoing research and enhanced public health strategies to mitigate its impact.

To provide further epidemiological evidence, elucidate the progress in disease health management, offer relevant message on disease focused interventions, and help different countries develop targeted strategies for prevention and control, a comprehensive and long-term analysis of PAH burden is necessary. In view of this, we conducted a comprehensive analysis using the Global Burden of Disease (GBD) 2021 dataset. Our objective was to evaluate the trends in PAH incidence, mortality, and disability-adjusted life years (DALYs) from 1990 to 2021 at global, regional, and national levels. In order to identify populations most affected by PAH and to encourage the development of appropriate prevention and treatment strategies, the studies were also stratified by age, gender, and Socio-Demographic Index (SDI).

## Methods

### Data acquisition and download

This study utilized data from the GBD 2021 study, which provides a wide-ranging assessment of health loss attributable to 371 diseases, injuries, and conditions, as well as 88 risk factors, across 204 countries and 811 subnational locations (12). The GBD 2021 database encompasses extensive epidemiological data, and employs advanced standardized methodologies to generate robust and comparable health estimates (12). In current study, we extracted data on the incidence, mortality, and DALYs related to PAH, as well as age, location, and sex-specific numbers and rates were calculated through the Global Health Data Exchange (GHDx) query tool.^1^ Notably, The indicators included in the GBD 2021 report were defined by estimates and their 95% uncertainty intervals (UIs)., which were defined by the 25th and 75th ordered values of 1,000 draw of the posterior distribution, and all ratios were reported by every 100,000 persons according to GBD’s algorithm.

The PAH-specific data, including the SDI, were obtained from online.^2^ The SDI scores range from 0 to 1 and represent a country’s level of social development, encompassing factors such as income per capita, educational attainment, and fertility rates (13).

### Case definition

Pulmonary hypertension is classified into 5 groups (5). In the GBD 2021, PAH was classified as Group 1 pulmonary hypertension, is a vascular disease characterized by pulmonary artery remodeling, elevated pulmonary pressures, and eventual right heart dysfunction.^3^ The GBD study defined PAH cases based on clinical diagnosis supported by evidence from either right heart catheterization or echocardiography. Cases identified through 10th iteration of the International Classification of Disease (ICD-10) codes 416 and I27.0 were included if diagnoses were confirmed via medical record review. To ensure specificity, ICD code I27.2 and data from ICD8, ICD9 BTL, and ICD10 tabulated formats were excluded due to their inability to reliably distinguish PAH from other pulmonary diseases. In countries where the introduction of I27.0 significantly altered PAH mortality trends, data before the implementation of I27.0 were excluded. This approach was designed to focus exclusively on PAH while excluding pulmonary hypertension groups 2–5, which have distinct pathophysiology and causes (14).

### Statistics analysis

Extensive prior research has thoroughly delineated the methodologies and protocols utilized in GBD studies (15). In this research, the burden of incidence, mortality, and DALYs during the study period was evaluated by age-standardized incidence rate (ASIR), age-standardized death rate (ASMR), age-standardized DALYs rate (ASDR), and estimated annual percentage changes (EAPC) from 1990 to 2021.

Estimated Annual Percentage Change (EAPC) is a widely used metric for examining the temporal trends in disease burden over specific periods. The EAPC was calculated from the formula: y = α + βx + ε, where y represents the natural logarithm of the age-standardized rate (ASR), and x denotes the calendar year. The EAPC is then calculated as 100 × [exp (β) – 1]. The presence of a positive EAPC with its 95% confidence intervals (CIs) is an indicator that there is an upward trend in ASR, whereas a negative EAPC and its corresponding 95% CIs suggest a downward trend. Using the EAPC values, hierarchical cluster analysis was conducted to assess the temporal patterns of disease burden changes across GBD regions, thereby identifying regions with similar trends (16). Additionally, given that the variables were not normally distributed. Smoothing splines models were used to evaluate the relationship between the burdens of age standardized rate and EAPC in 204 countries and territories. We fitted smooth splines using the Locally Weighted Scatterplot Smoothing (LOWESS) method, which automatically determines the degree, number, and location of nodes (knots) on the basis of the data and the span parameter (17).

The association between the burden of PAH and the SDI across 21 regions and 204 countries or territories was analyzed using smoothing spline models. Expected values were derived based on the SDI and age-standardized rates across all locations. To further assess this relationship, Locally Weighted Scatterplot Smoothing (LOWESS) and Spearman correlation were applied to calculate the R indices and *p*-values, capturing the strength and significance of the correlation between age-standardized rates and SDI (18).

Additionally, subgroup analyses were performed for specific age groups to identify differential trends across different demographics. Previous studies have demonstrated that BAPC model outperforms alternative prediction models in terms of coverage and accuracy, thereby offering enhanced reliability and precision in statistical estimation. All statistical analyses were performed using R software (version 4.4.1). All hypothesis tests were two-tailed with a significance level of *p* < 0.05.

### Ethical considerations

As this study utilized publicly available, de-identified data from the GBD 2021 study, ethical approval was not required. However, all analyses were conducted in accordance with relevant ethical guidelines and data usage policies.

## Results

### Global level

**Figure 1:**
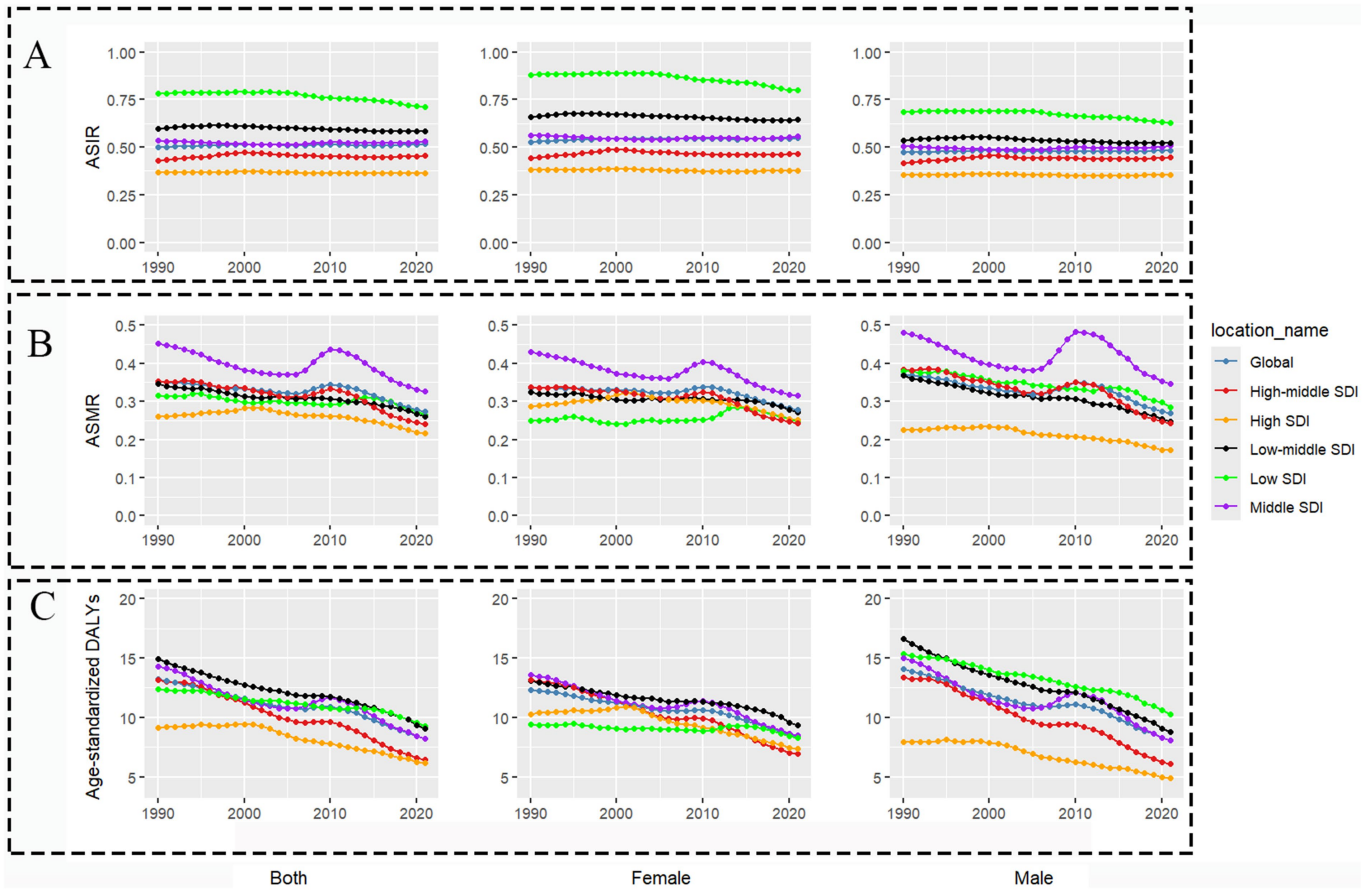
Trends in pulmonary arterial hypertension ASIR, ASMR and ASDRfrom 1990 to 2021. **(A)** ASIR. **(B)** ASMR. **(C)** ASDR. ASIR, age-standardized incidence rate; ASMR, age-standardized death rate; DALYs, disability-adjusted life years; ASDR, age-standardized DALYs rate.

The global burden of PAH remained considerable, with the number of incident cases of PAH increased by 85.6%, from 23,301 (95% UI, 19,037-27,809) in 1990 to 43,251 (95% UI, 34,705-52,441) in 2021 (Table 1). The ASIR showed a slight increase from 0.50 per 100,000 persons in 1990 to 0.52 per 100,000 population in 2021, with an EAPC of 0.05 (95% CI: 0.03–0.07), indicating a stable trend (Table 1; Figure 1A). Globally, PAH-related deaths increased from 14,842 (95% UI: 12,370–17,485) in 1990 to 22,021 (95% UI: 18,239–25,352) in 2021 (Table 2). The age-standardized mortality rate (ASMR) was 0.27 per 100,000 persons (95% UI: 0.23–0.32), with an EAPC of −0.57 (95% CI: −0.72 to −0.42), indicating a decreasing trend in ASMR (Table 2; Figure 1B). In 2021, PAH accounted for 642,104 DALYs (95% UI: 552,273–728,993), with an ASDR of 8.24 per 100,000 persons (95% UI: 7.14–9.39), with an EAPC of −1.31 (95% CI: −1.43 to −1.19), reflecting a decrease in ASDR (Table 3; Figure 1C).

**Table 1:** Incidence of Pulmonary Arterial Hypertension between 1990 and 2021 at the global and regional level.

Location	All-age cases (95% UI)		ASIR (95% UI)		EAPC_95CI 1990–2021
	1990	2021	1990	2021	
Global	23,301 (19,037, 27,809)	43,251 (34,705, 52,441)	0.5 (0.4, 0.6)	0.52 (0.42, 0.62)	0.05 (0.03 to 0.07)
High-middle SDI	4,469 (3,599, 5,419)	7,734 (6,187, 9,586)	0.43 (0.35, 0.52)	0.46 (0.37, 0.55)	0.03 (−0.05 to 0.12)
Low SDI	2,749 (2,266, 3,274)	5,712 (4,685, 6,847)	0.78 (0.64, 0.93)	0.71 (0.58 0.85)	−0.3 (−0.36 to −0.25)
High SDI	3,677 (2,945, 4,509)	5,612 (4,500, 6,959)	0.37 (0.3, 0.45)	0.37 (0.29, 0.44)	−0.06 (−0.09 to −0.04)
Low-middle SDI	5,120 (4,183, 6,102)	9,984 (8,086, 12,020)	0.6 (0.48, 0.71)	0.59 (0.47, 0.7)	0.03 (−0.05 to 0.12)
Middle SDI	7,264 (5,922, 8,765)	14,174 (11,319, 17,383)	0.54 (0.43, 0.64)	0.53 (0.43, 0.64)	−0.17 (−0.2 to −0.13)
Andean Latin America	151 (124, 182)	347 (282, 418)	0.53 (0.43, 0.63)	0.33 (0.26, 0.4)	0.14 (0.05 to 0.22)
Australasia	77 (62, 94)	153 (123, 190)	0.35 (0.28, 0.43)	0.44 (0.36, 0.54)	0.18 (0.14 to 0.21)
Caribbean	137 (112, 166)	247 (199, 299)	0.45 (0.37, 0.55)	0.5 (0.4, 0.6)	0.27 (0.19 to 0.36)
Central Asia	233 (190, 279)	399 (320, 487)	0.41 (0.33, 0.5)	0.56 (0.45, 0.67)	0.09 (−0.01 to 0.18)
Central Europe	537 (430, 657)	794 (644, 986)	0.39 (0.31, 0.47)	0.58 (0.47, 0.69)	0.4 (0.28 to 0.53)
Central Latin America	679 (556, 818)	1,267 (1,024, 1,535)	0.56 (0.46, 0.68)	0.37 (0.3, 0.45)	−0.23 (−0.39 to −0.07)
Central Sub-Saharan Africa	302 (247, 363)	776 (636, 938)	0.81 (0.65, 0.97)	0.48 (0.39, 0.58)	0.27 (0.1 to 0.44)
East Asia	5,295 (4,275, 6,415)	9,572 (7,599, 11,898)	0.51 (0.41, 0.61)	0.47 (0.38, 0.57)	−0.12 (−0.16 to −0.07)
Eastern Europe	941 (757, 1,162)	1,198 (962,1,488)	0.37 (0.3, 0.45)	0.43 (0.35, 0.52)	0.22 (−0.07 to 0.5)
Eastern Sub-Saharan Africa	1,251 (1,036, 1,497)	2,687 (2,209, 3,243)	0.99 (0.81, 1.17)	0.41 (0.33, 0.49)	−0.07 (−0.15 to 0.01)
High-income Asia Pacific	595 (475, 733)	942 (761, 1,175)	0.31 (0.25, 0.37)	0.55 (0.44, 0.66)	0.18 (0.14 to 0.22)
High-income North America	845 (683, 1,031)	1,524 (1,209, 1,891)	0.27 (0.22, 0.33)	0.49 (0.4, 0.59)	0.35 (0.27 to 0.42)
North Africa and Middle East	1,353 (1,106, 1,612)	2,881 (2,326, 3,524)	0.56 (0.45, 0.67)	0.33 (0.27, 0.41)	−0.45 (−0.62 to −0.28)
Oceania	26 (21, 31)	65 (53, 79)	0.57 (0.46, 0.68)	0.49 (0.39, 0.59)	−0.03 (−0.12 to 0.07)
South Asia	4,573 (3,718, 5,444)	9,520 (7,651, 11,461)	0.56 (0.46, 0.67)	0.52 (0.42, 0.63)	−0.07 (−0.1 to −0.04)
Southeast Asia	1,922 (1,567, 2,311)	4,082 (3,268, 4,966)	0.55 (0.44, 0.66)	0.3 (0.24, 0.37)	0.08 (0.03 to 0.13)
Southern Latin America	162 (132, 198)	259 (208, 320)	0.34 (0.28, 0.41)	0.61 (0.49, 0.73)	0.02 (−0.02 to 0.06)
Southern Sub-Saharan Africa	317 (260,3 78)	537 (439, 649)	0.81 (0.67, 0.97)	0.83 (0.67, 0.98)	−0.08 (−0.17 to 0.01)
Tropical Latin America	602 (488, 725)	1,226 (984, 1,492)	0.5 (0.4, 0.6)	0.92 (0.75, 1.09)	−0.07 (−0.19 to 0.06)
Western Europe	2,176 (1,739, 2,656)	2,593 (2,092, 3,209)	0.46 (0.37, 0.55)	0.75 (0.61, 0.9)	−0.42 (−0.48 to −0.35)
Western Sub-Saharan Africa	1,128 (931, 1,343)	2,181 (1,786, 2,634)	0.83 (0.68, 0.99)	0.64 (0.52, 0.78)	−1.15 (−1.27 to −1.02)

ASIR, Age standardized incidence rate; EAPC, estimated annual percentage change; SDI, socio-demographic index; 95% UI, 95% uncertainty interval; 95% CI, 95% confidence interval.

**Table 2:** Morality of Pulmonary Arterial Hypertension between 1990 and 2021 at the global and regional level.

Location	Death cases (95% UI)		ASMR (95% UI)		EAPC_95CI 1990–2021
	1990	2021	1990	2021	
Global	14,842 (12,370, 17,485)	22,021 (18,239, 25,352)	0.35 (0.29, 0.42)	0.27 (0.23, 0.32)	−0.57 (−0.72 to −0.42)
High-middle SDI	3,214 (2,772, 3,908)	4,326 (3,594, 5,141)	0.35 (0.31, 0.43)	0.24 (0.2, 0.29)	−1.07 (−1.27 to −0.87)
Low SDI	1,145 (740, 1,755)	1,782 (1,147, 2,544)	0.32 (0.15, 0.51)	0.27 (0.15, 0.4)	−0.33 (−0.44 to −0.22)
High SDI	2,617 (2,379, 2,850)	4,621 (3,919, 5,054)	0.26 (0.24, 0.28)	0.22 (0.19, 0.23)	−0.56 (−0.74 to −0.38)
Low-middle SDI	3,125 (2,220, 3,904)	3,728 (2,757, 5,091)	0.35 (0.22, 0.47)	0.26 (0.18, 0.38)	−0.71 (−0.79 to −0.63)
Middle SDI	4,729 (3,774, 5,852)	7,548 (5,141, 9,026)	0.45 (0.35, 0.58)	0.33 (0.22, 0.39)	−0.63 (−0.88 to −0.37)
Andean Latin America	84 (56, 112)	91 (72, 119)	0.28 (0.21, 0.35)	0.16 (0.12, 0.2)	−1.7 (−1.97 to −1.44)
Australasia	45 (39, 57)	58 (49, 65)	0.21 (0.18, 0.26)	0.11 (0.1, 0.13)	−2.06 (−2.49 to −1.62)
Caribbean	124 (83, 169)	95 (66, 130)	0.38 (0.28, 0.49)	0.2 (0.13, 0.29)	−2.53 (−2.78 to −2.29)
Central Asia	208 (163, 240)	319 (261, 382)	0.4 (0.31, 0.47)	0.41 (0.34, 0.48)	0.3 (0.06 to 0.53)
Central Europe	355 (309, 394)	438 (398, 479)	0.26 (0.22, 0.28)	0.21 (0.19, 0.23)	−0.71 (−0.97 to −0.45)
Central Latin America	180 (157, 210)	201 (177, 230)	0.16 (0.14, 0.19)	0.08 (0.07, 0.1)	−2.54 (−2.89 to −2.19)
Central Sub-Saharan Africa	87 (55, 163)	131 (62, 237)	0.24 (0.11, 0.47)	0.19 (0.08, 0.37)	−0.67 (−0.74 to −0.61)
East Asia	4,115 (3,141, 5,526)	7,490 (4,986, 9,266)	0.59 (0.45, 0.81)	0.41 (0.28, 0.5)	−0.57 (−0.94 to −0.19)
Eastern Europe	563 (512, 651)	278 (258, 300)	0.24 (0.22, 0.27)	0.09 (0.08, 0.1)	−3.78 (−4.18 to −3.37)
Eastern Sub-Saharan Africa	366 (217, 687)	468 (219, 878)	0.27 (0.12, 0.52)	0.18 (0.07, 0.34)	−1.46 (−1.51 to −1.42)
High-income Asia Pacific	434 (410, 459)	1,049 (826, 1,201)	0.26 (0.24, 0.27)	0.23 (0.2, 0.26)	−0.46 (−0.59 to −0.33)
High-income North America	1,064 (947, 1,167)	1,880 (1,620, 2,043)	0.32 (0.28, 0.35)	0.29 (0.26, 0.31)	−0.45 (−0.61 to −0.29)
North Africa and Middle East	2,142 (1,309, 2,739)	1,896 (1,328, 2,305)	0.77 (0.56, 1)	0.44 (0.31, 0.53)	−1.36 (−1.5 to −1.21)
Oceania	12 (8, 19)	25 (17, 43)	0.28 (0.18, 0.53)	0.24 (0.16, 0.48)	−0.54 (−0.59 to −0.49)
South Asia	2,385 (1,502, 3,418)	3,549 (2,321, 5,532)	0.31 (0.17, 0.5)	0.25 (0.16, 0.42)	−0.48 (−0.58 to −0.38)
Southeast Asia	506 (340, 1,094)	741 (525, 1,850)	0.15 (0.09, 0.43)	0.12 (0.08, 0.32)	−0.7 (−0.78 to −0.62)
Southern Latin America	169 (151, 186)	150 (138, 162)	0.36 (0.32, 0.4)	0.18 (0.17, 0.2)	−2.06 (−2.25 to −1.87)
Southern Sub-Saharan Africa	43 (32, 58)	72 (53, 86)	0.12 (0.08, 0.17)	0.11 (0.08, 0.13)	−0.03 (−0.26 to 0.2)
Tropical Latin America	394 (373, 412)	779 (714, 822)	0.37 (0.35, 0.39)	0.32 (0.29, 0.34)	−0.55 (−1.08 to −0.02)
Western Europe	1,233 (1,094, 1,380)	1,788 (1,533, 1,943)	0.24 (0.21, 0.27)	0.18 (0.16, 0.19)	−0.76 (−1.26 to −0.25)
Western Sub-Saharan Africa	335 (195, 616)	523 (306, 774)	0.25 (0.09, 0.51)	0.17 (0.07, 0.28)	−1.38 (−1.5 to −1.27)

ASMR, Age standardized death rate; EAPC, estimated annual percentage change; SDI, socio-demographic index; 95% UI, 95% uncertainty interval; 95% CI, 95% confidence interval.

**Table 3:** Disability adjusted life years of Pulmonary Arterial Hypertension between 1990 and 2021 at the global and regional level.

Location	DALYs (95% UI)		Age-standardized DALY rate (95% UI)		EAPC_95CI 1990–2021
	1990	2021	1990	2021	
Global	687,419 (535,241, 813,086)	642,104 (552,273, 728,993)	13.21 (10.78, 15.36)	8.24 (7.14, 9.39)	−1.31 (−1.43 to −1.19)
High-middle SDI	127,638 (106,711, 154,438)	99,448 (85,757, 117,639)	13.14 (10.91, 16.04)	6.48 (5.61, 7.87)	−2.2 (−2.36 to −2.05)
Low SDI	71,125 (48,628, 111,614)	95,342 (67,471, 133,050)	12.42 (7.78, 19.19)	9.3 (6.08, 13.2)	−0.78 (−0.85 to −0.71)
High SDI	81,792 (77,185, 88,110)	93,182 (84,873, 99,192)	9.16 (8.71, 9.9)	6.16 (5.76, 6.49)	−1.39 (−1.57 to −1.22)
Low-middle SDI	195,281 (117,185, 245,591)	156,400 (122,426, 194,166)	14.92 (10.74, 18.41)	9.07 (7.05, 11.6)	−1.33 (−1.43 to −1.24)
Middle SDI	210,946 (172,857, 258,349)	197,171 (148,781, 232,321)	14.29 (11.66, 17.69)	8.23 (6.26, 9.7)	−1.4 (−1.61 to −1.18)
Andean Latin America	5,049 (2,977, 7,422)	3,544 (2,795, 4,490)	11.92 (7.83, 16.43)	5.73 (4.52, 7.28)	−2.11 (−2.35 to −1.86)
Australasia	1,514 (1,337, 1,856)	1,434 (1,305, 1,563)	7.37 (6.49, 9.06)	3.67 (3.38, 3.99)	−2.33 (−2.8 to −1.86)
Caribbean	7,398 (4,170, 11,145)	5,071 (2,988, 7,877)	19.72 (11.78, 28.82)	11.73 (6.48, 18.74)	−2.01 (−2.19 to −1.83)
Central Asia	9,071 (7,195, 10,646)	11,619 (9,514, 14,202)	14.14 (11.27, 16.45)	12.91 (10.61, 15.6)	−0.33 (−0.55 to −0.11)
Central Europe	11,026 (9,784, 12,154)	10,424 (9,512, 11,459)	8.15 (7.25, 8.95)	6.05 (5.5, 6.67)	−1.01 (−1.25 to −0.78)
Central Latin America	9,966 (8,844, 11,855)	7,246 (6,391, 8,407)	6.12 (5.4, 7.16)	3 (2.63, 3.51)	−2.67 (−2.99 to −2.35)
Central Sub-Saharan Africa	5,367 (3,258, 10,525)	6,524 (3,586, 11,025)	9.09 (5.65, 17.13)	6.16 (2.94, 11.09)	−1.14 (−1.24 to −1.05)
East Asia	151,596 (117,394, 205,773)	154,740 (102,939, 190,399)	15.78 (12.34, 21.21)	8.84 (5.99, 11.01)	−1.32 (−1.68 to −0.97)
Eastern Europe	20,628 (18,781, 23,860)	8,357 (7,760, 8,996)	9.23 (8.47, 10.49)	3.32 (3.1, 3.56)	−3.99 (−4.39 to −3.58)
Eastern Sub-Saharan Africa	23,148 (14,694, 45,765)	26,605 (14,265, 49,615)	11.11 (6.18, 21.17)	6.85 (3.3, 12.62)	−1.62 (−1.67 to −1.58)
High-income Asia Pacific	18,474 (17,689,1 9,467)	19,988 (17,442, 21,997)	12.03 (11.43, 12.79)	8.31 (7.74, 8.87)	−1.5 (−1.7 to −1.29)
High-income North America	30,206 (27,736, 32,715)	38,373 (35,060, 40,844)	10.13 (9.39, 10.99)	7.71 (7.17, 8.18)	−1.14 (−1.32 to −0.95)
North Africa and Middle East	145,728 (74,879, 204,070)	80,753 (58,086, 98,810)	35.84 (21.25, 46.14)	14.81 (10.76, 17.96)	−2.43 (−2.57 to −2.29)
Oceania	704 (448, 1,090)	1,455 (988,2,411)	10.85 (7.02, 17.55)	10.14 (6.9, 17.1)	−0.22 (−0.26 to −0.17)
South Asia	136,086 (77,808, 184,381)	136,563 (97,809, 189,353)	12.24 (7.79, 17.12)	8.54 (6.02, 12.46)	−0.95 (−1.03 to −0.87)
Southeast Asia	27,786 (18,242, 51,943)	31,112 (22,913, 58,708)	6.31 (4.28, 12.72)	4.65 (3.39, 9.25)	−0.92 (−1 to −0.84)
Southern Latin America	7,983 (7,181, 8,803)	4,739 (4,439, 5,092)	16.28 (14.65, 17.95)	6.55 (6.13, 7.07)	−2.87 (−3.01 to −2.72)
Southern Sub-Saharan Africa	2,188 (1,667, 2,875)	3,212 (2,359, 3,891)	4.7 (3.65, 6.37)	4.33 (3.21, 5.2)	−0.02 (−0.26 to 0.21)
Tropical Latin America	19,065 (17,856, 20,357)	24,235 (23,002, 2 5,403)	14.18 (13.35, 14.98)	10.22 (9.65, 10.78)	−1.12 (−1.66 to −0.59)
Western Europe	34,599 (31,743, 38,281)	34,043 (31,024, 36,440)	8.27 (7.69,9.18)	5.05 (4.75, 5.32)	−1.55 (−1.99 to −1.11)
Western Sub-Saharan Africa	19,834 (13,553, 38,853)	32,071 (22,084, 47,222)	9.34 (5.22, 16.94)	6.5 (3.77, 9.47)	−1.14 (−1.27 to −1.01)

ASDR, Age standardized disability adjusted life years rate; EAPC, estimated annual percentage change; SDI, socio-demographic index; 95% UI = 95% uncertainty interval; 95% CI, 95% confidence interval.

### Regional level

The global burden of PAH exhibits notable variations that are closely associated with SDI levels across regions. At the SDI region level, the ASIR exhibited notable inconsistencies. From 1990 to 2021, low SDI regions had the highest ASIR decreasing from 0.78 to 0.71 per 100,000 persons, with an EAPC of −0.3% (95% CI: −0.36% to −0.25%), representing the most substantial decrease (Table 1; Figure 1A). In contrast, ASIR increased in the low-middle and high-middle SDI regions, while it decreased was observed in the remaining three regions. High SDI regions exhibited the lowest ASMR and ASDR, whereas low SDI regions recorded the highest ASDR (Table 3). Specifically, middle SDI regions reported the highest ASMR at 0.33 (95% UI: 0.22–0.39) compared to 0.22 per 100,000 persons (95% UI: 0.19–0.23) in high SDI regions (Table 2). Conversely, the ASDR was 9.3 per 100,000 persons (95% UI: 6.08–13.2) in high-middle SDI regions and markedly lower in high SDI regions at 6.16 per 100,000 persons (95% UI: 5.76–6.49) (Table 3).

**Figure 2:**
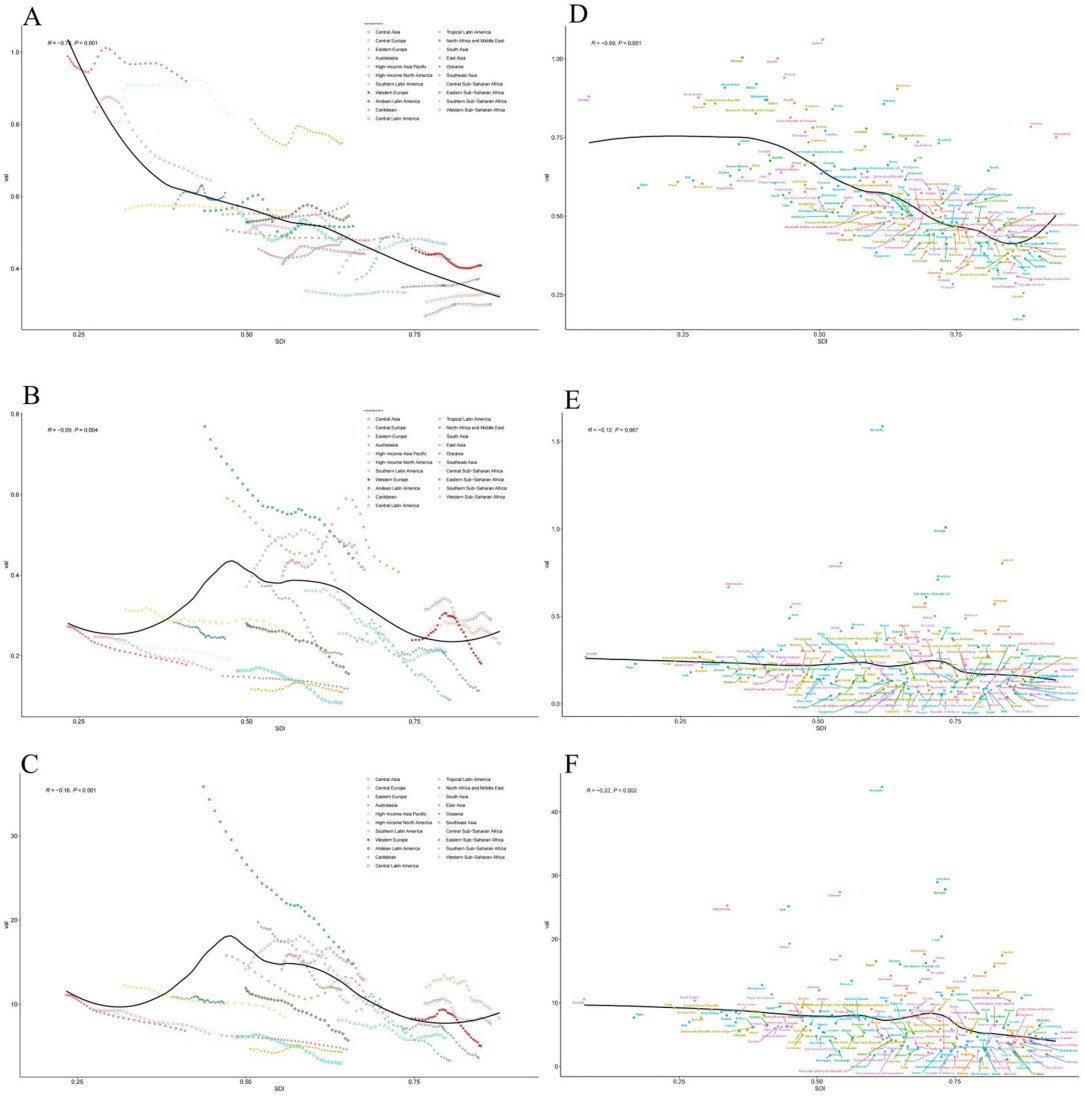
Age standardized rates of PAH among regions and nations based on SDI in 2021. **(A)** ASIR in 21 regions. **(B)** ASMR in 21 regions. **(C)** ASDRin 21 regions. **(D)** T ASIR in 204 countries. **(E)** ASMR in 204 countries. **(F)** ASDR in 204 countries. SDI, Social-Demographic Index; ASIR, age-standardized incidence rate; DALYs, disability-adjusted life years; ASDR, age-standardized DALYs rate; ASMR, age-standardized death rate.

The fitted curve revealed a nonlinear relationship between the SDI and both ASMR and ASDR, characterized by a left-skewed inverted U-shaped, peaking at low SDI levels. The burden of PAH exhibited a marked increase in regions with low SDI (SDI < 0.4). In contrast, regions with higher SDI experienced a gradual decrease in PAH burden as SDI improved (Figures 2B,C).

**Figure 3:**
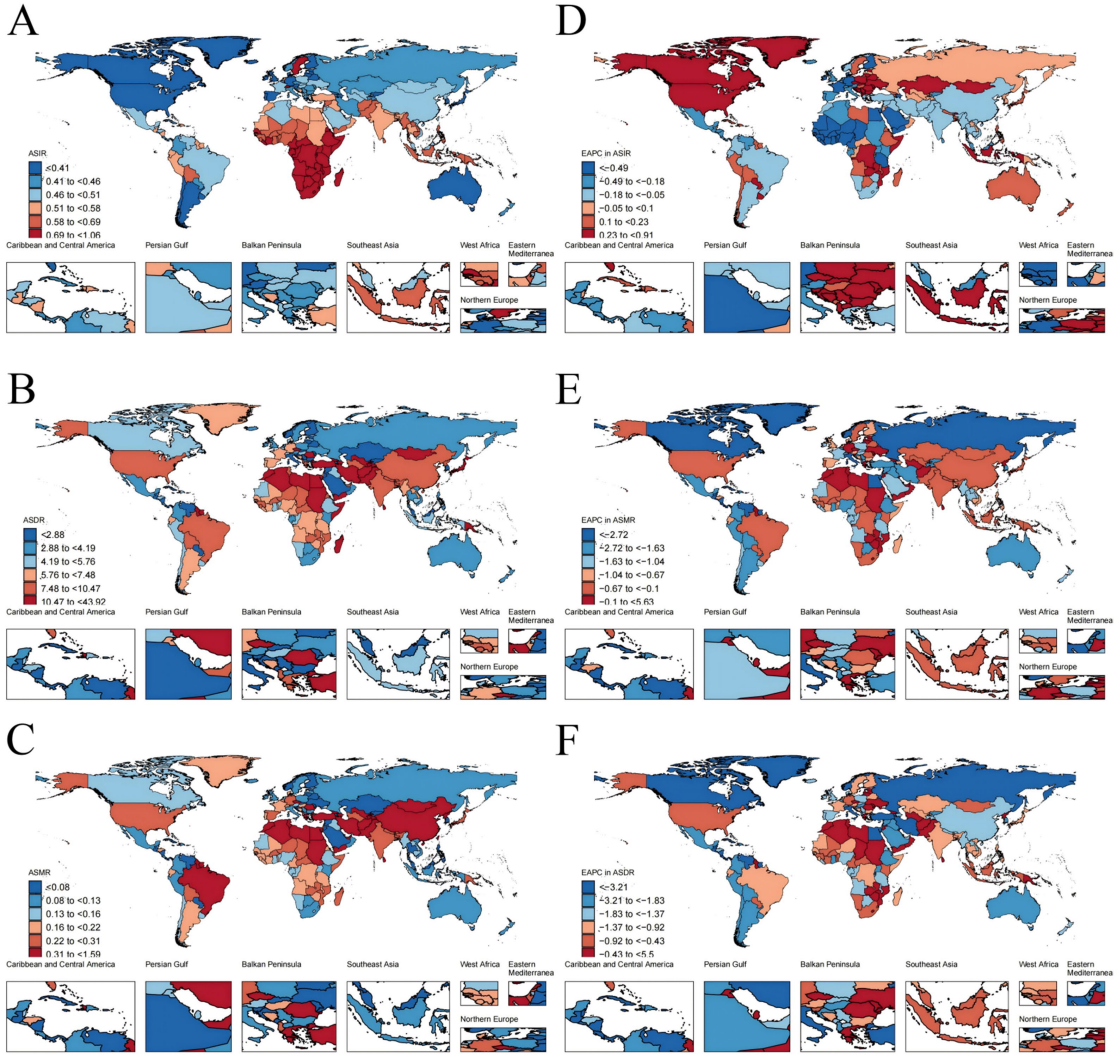
The global disease burden of Pulmonary Arterial Hypertension in 204 countries. **(A)** ASIR. **(B)** ASMR. **(C)** ASDR. **(D)** EAPC in ASIR. **(E)** EAPC in ASMR. **(F)** EAPC in ASDR. ASIR, age-standardized incidence rate; ASMR, age-standardized death rate; DALYs, disability-adjusted life years; ASDR, age-standardized DALYs rate; EAPC, estimated annual percentage change.

**Figure 4:**
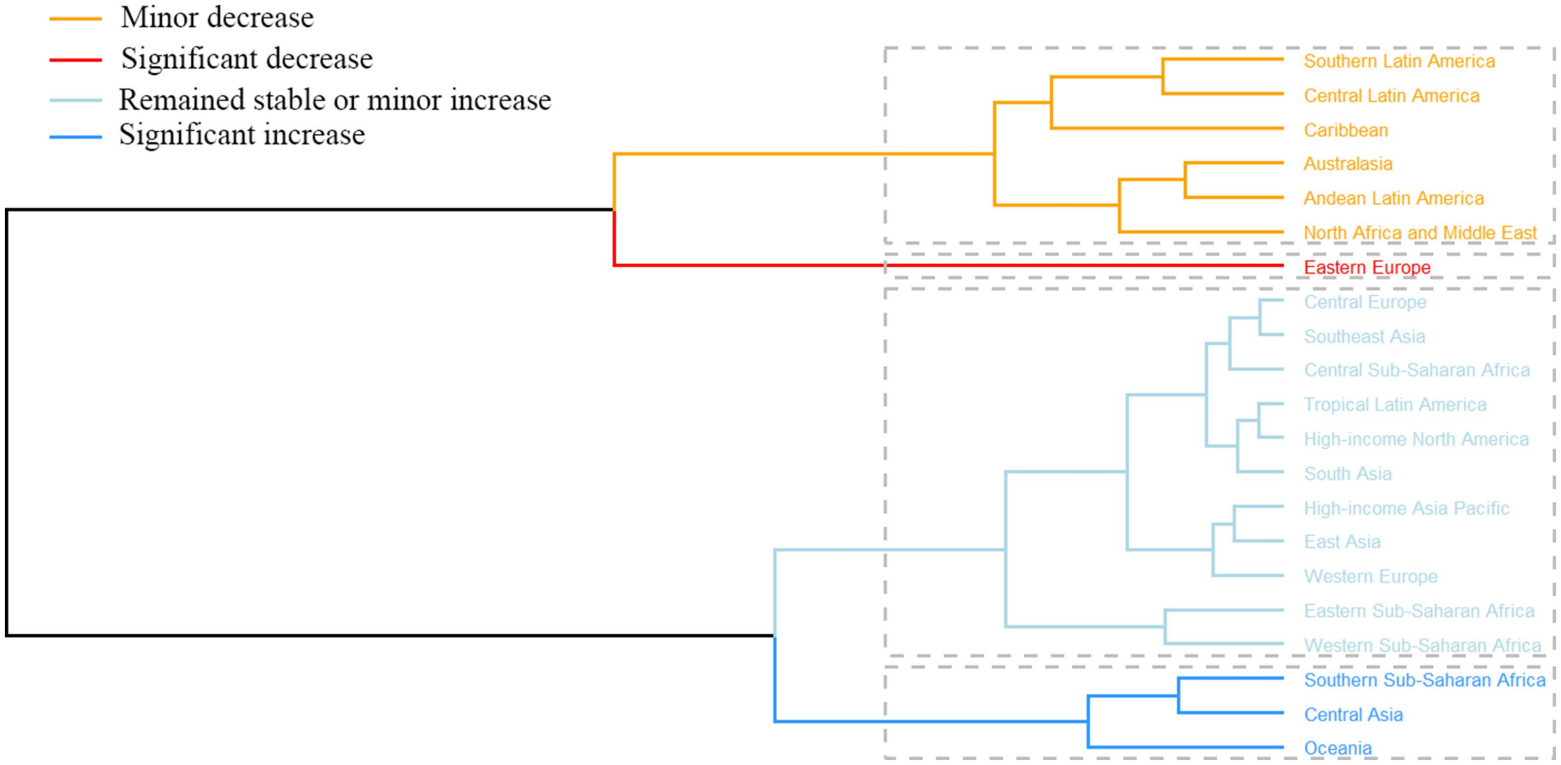
Results from clustering EAPC values for age-standardized death and DALY rates associated with PAH between 1990 and 2021. EAPC, estimated annual percentage change; PAH, pulmonary arterial hypertension; DALYs, disability-adjusted life years.

Across the 21 GBD regions, Sub-Saharan Africa bears the highest burden of PAH incidence globally, with the top four incidence rates observed within this region. Specifically, the ASIR was highest in Southern Sub-Saharan Africa, reaching 0.92 per 100,000 persons (95% UI: 0.75–1.09), followed closely by Central Sub-Saharan Africa at 0.82 per 100,000 persons (95% UI: 0.67–0.98) (Table 1; Figure 3A). However, the most pronounced decline in ASIR occurred in Western sub-Saharan Africa (EAPC −1.15, 95%CI: −1.27 to −1.02) (Table 1; Figure 3D). Central Asia and Middle East, North Africa and Middle East and East Asia exhibited significanely high ASMRs for PAH, each exceeding 0.4 per 100,000 persons (Table 2; Figure 3B). From1990 to 2021, the ASMR of PAH increased most in Central Asia (EAPC 0.3, 95% UI: 0.06 to 0.53) and decreased most in Eastern Europe (EAPC -3.78, 95% UI: −4.18 to −3.37) (Table 2; Figure 3E). Furthermore, Central Asia was the only region showing an increasing trend. Similarly, the regions with the highest ASDR were North Africa and the Middle East (14.81 per 100,000 persons; 95% UI: 10.76–17.95), Central Asia (12.91 per 100,000 persons; 95% UI: 10.61–15.60), and the Caribbean (11.73 per 100,000 persons; 95% UI: 6.48–18.74) (Table 3; Figure 3C). From 1990 to 2021, ASDR for PAH noticeably decreased across all regions, with the most significant decrease observed in Eastern Europe (EAPC -3.99, 95% CI: −4.39 to −3.58) (Table 3; Figure 3F). Hierarchical clustering analysis revealed a marked increase age-standardized rates for deaths and DALYs was observed in Central Asia, Southern Sub-Saharan Africa, Oceania (Figure 4; Supplementary Figure 1).

### National level

In 2021, Zambia, Ethiopia, and Uganda exhibit the highest ASIR of PAH, with rates of 1.06, 1.00, and 1.00 per 100,000 persons, respectively (Figure 3 (A)). In 2021, countries most affected by PAH in terms of ASMR and DALYs include Mongolia, Georgia, Tajikistan, and Mauritius. Mongolia leads in both metrics, holding the top ASMR (1.59 per 100,000 persons) and DALYs rate (43.92 per 100,000 persons). Georgia ranks second in ASMR (1.00 per 100,000 persons) and third in DALYs (27.83 per 100,000 persons). Tajikistan is third in ASMR (0.81 per 100,000 persons) and fourth in DALYs (27.43 per 100,000 persons). Mauritius ranks fifth in ASMR (0.71 per 100,000 persons) and second in DALYs (28.96 per 100,000 persons).

From 1990 to 2021, Slovakia saw the largest increase in ASIR (EAPC 0.91%), while Burkina Faso experienced the greatest decrease (EAPC -1.99%). Latvia recorded the highest rise in ASMR (EAPC 5.63%). Conversely, Puerto Rico demonstrated the most significant decreases in both ASMR (EAPC -6.64%) and DALYs (EAPC -6.72%). In contrast, Mauritius showed the largest increase in DALYs (EAPC 5.5%) (Figures 3B,C).

### Age and sex patterns

**Figure 5:**
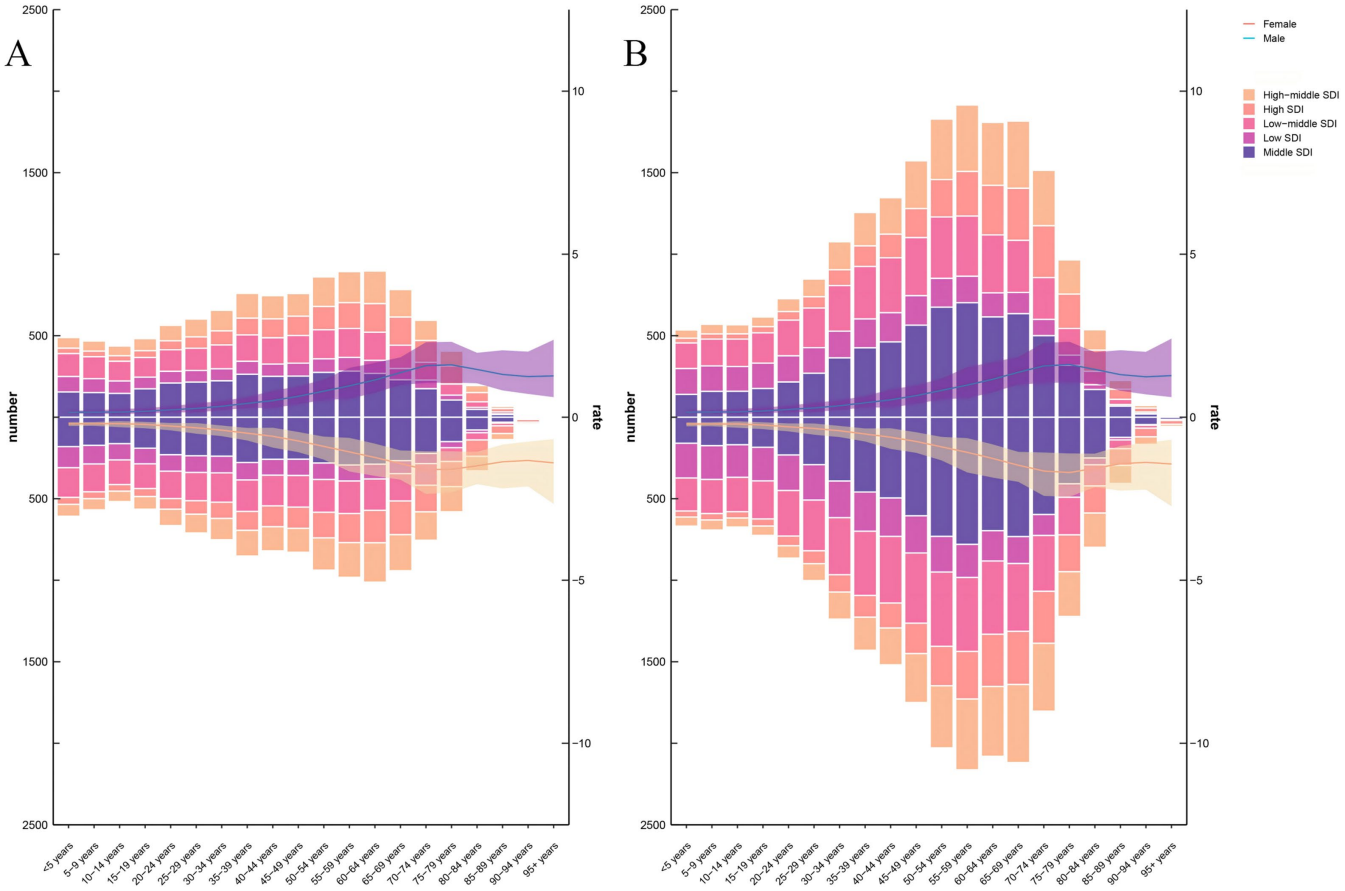
The age-specific numbers and ASIRs of PAH by SDI regions in 1990 and 2021. **(A)** ASIR in 1990. **(B)** ASIR in 2021.ASIR, age-standardized incidence rate; PAH, pulmonary arterial hypertension; SDI, Social-Demographic Index.

Between 1990 and 2021, global patterns of incidence, mortality, and DALYs have significant transformed across various age groups, genders, and SDI levels. In 1990, lower SDI regions experienced high incidence rates, particularly among children under five (Figure 5A). By 2021, incidence rates in younger populations markedly decreased, reflecting improvements in healthcare infrastructure, vaccination programs, and disease prevention initiatives. Conversely, incidence has surged among older adults, especially those aged 50 and above, in high and high-middle SDI regions (Figure 5B).

In 1990, mortality rates were predominantly high among younger populations in low SDI regions. By 2021, mortality rates for children under five has significantly decreased. In contrast, mortality rates among older adults, particularly those aged 65 and above, have increased in high SDI regions. (Supplementary Figures 2A,B). In 1990, DALYs were disproportionately high among younger populations in low SDI regions, where infectious diseases and child mortality were predominant health issues. By 2021, DALYs in younger age groups had substantially decreased, particularly in lower SDI regions. However, the distribution of DALYs has shifted toward older populations in high and high-middle SDI regions (Supplementary Figures 3A,B).

### The influential factors for EAPC

**Figure 6:**
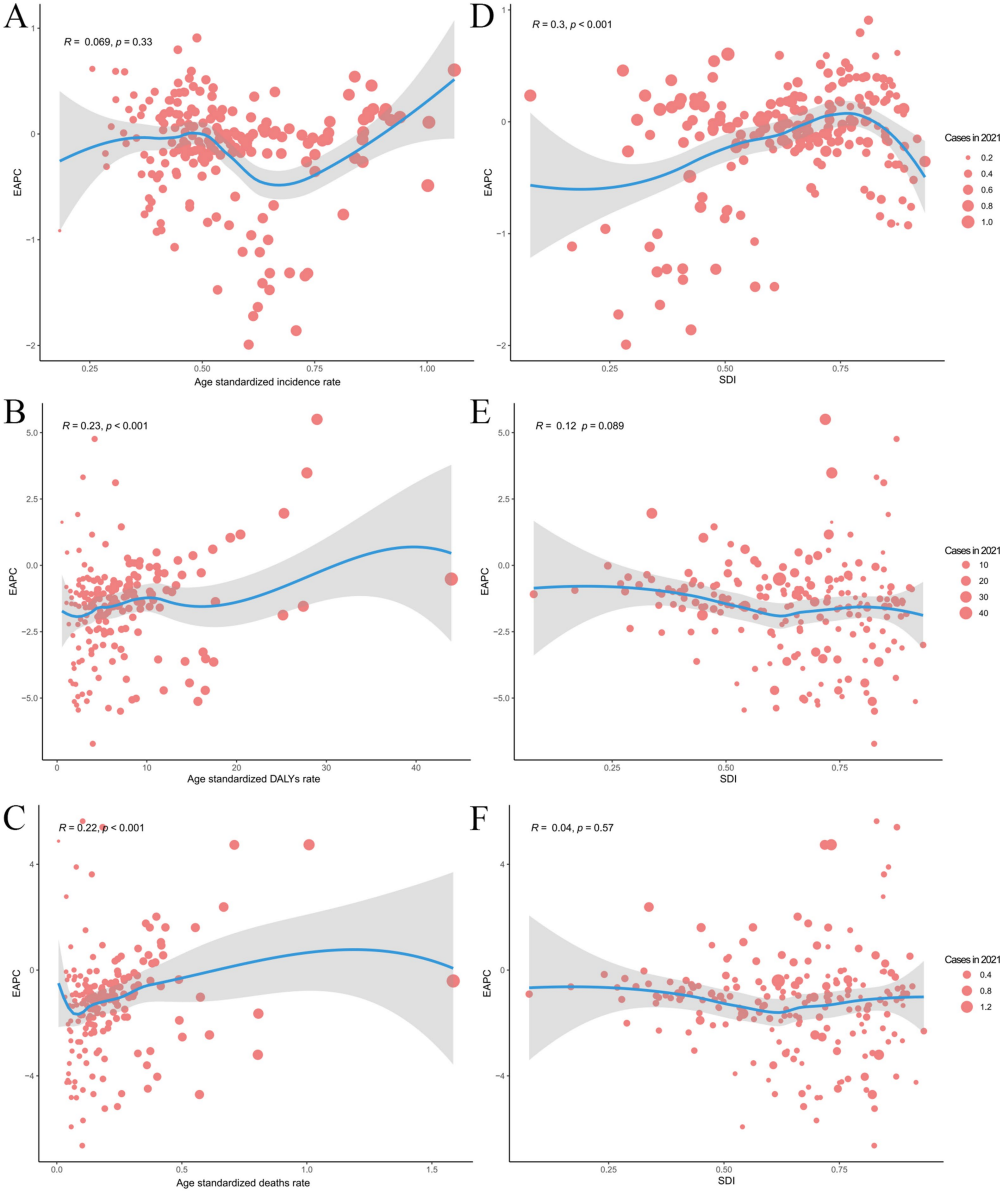
Correlation between EAPC and myocarditis ASIR, ASMR and Age-standardized disability-adjusted life-years in 1990 and SDI in 2021. **(A)** EAPC in ASIR in 1990. **(B)** EAPC in ASMR in 1990. **(C)** EAPC in ASDR in 1990. **(D)** EAPC in SDI in 2021. **(E)** EAPC in SDI in 2021. **(F)** EAPC in SDI in 2021. EAPC, estimated annual percentage change; ASIR, age-standardized incidence rate; ASMR, age-standardized death rate; SDI, Social-Demographic Index.

We analyzed the correlation coefficients between the EAPC and ASR in 1990 (Figures 6A–C), as well as the SDI in 2021 (Figures 6D–F). Results show a positive correlation between EAPC and both ASMR (R = 0.22, *p* < 0.001) (Figure 6C) and ASDR (R = 0.23, p < 0.001) in 1990 (Figure 6B). Additionally, there was a slight positive correlation between EAPCs and SDI (ASIR: R = 0.3, *p* < 0.001) (Figure 6D). Furthermore, countries with a higher SDI showed an increasing trend in 2021, reflecting patterns previously observed across diverse SDI regions.

## Discussion

Previous studies have attempted to quantify the disease burden associated with PAH; however, most investigations have been limited to specific regions or countries (19, 20). Only a few studies have explored this issue on a global scale. Additionally, cohort studies confined to local populations may suffer from methodological limitations that compromise their precision. In contrast, the GBD 2021 study utilized diverse data sources, including household surveys, vital statistics, and various databases, and encompassed a wider range of countries. This approach provides more comprehensive and accurate estimates of the disease burden. Our findings offer valuable insights into the evolving burden of PAH over the past 32 years across regions and countries with different income levels. From 1990 to 2021, the burden of PAH has increased in several regions and nations globally.

In terms of age and sex distribution, this research reveals that from 1990 to 2021, there was an increasing trend in death and DALY due to PAH globally. The disease burden was more pronounced among children under five, as well as the middle-aged and older adult population. High SDI regions, having made substantial progress in reducing infectious diseases and child mortality, now face an increasing burden of chronic diseases among older adults. In contrast, lower SDI regions, despite improvements in child health, continue to grapple with managing both infectious diseases and the rising incidence of chronic conditions. In summary, significant progress has been achieved in reducing incidence, mortality, and DALYs among younger populations in global, particularly children under five. However, in high SDI regions, the global health burden has increasingly shifted toward older adults. This increase is primarily attributed to the increased prevalence of non-communicable diseases such as cardiovascular disease, cancer, and diabetes, which are more common in aging populations (21, 22). This shift underscores the growing impact of chronic, age-related diseases and highlights the need for healthcare systems to adapt to the evolving demands of aging populations.

PAH has a higher prevalence in women, who also demonstrate improved prognosis, potentially attributable to oestrogen-mediated mechanisms (23, 24). Despite the overall increasing global burden of PAH over the past 32 years, mortality rates, particularly among women, have risen. Conversely, the ASMR and ASDR have shown a decreasing trend. Where chronic, non-communicable diseases significantly contribute to both morbidity and mortality. Additionally, women tend to experience higher DALYs in older age groups due to longer life expectancy and greater exposure to chronic illnesses (25). In conclusion, the gender disparity in the disease burden associated with PAH remains unclear, and the underlying mechanisms have yet to be fully elucidated (26). Therefore, comprehensive investigations are urgently needed to clarify these differences.

In terms of inter-country inequality, the disease distribution map and cross-regional comparisons indicate that the burden of PAH is closely associated with the SDI (27). This pattern likely reflects a multifaceted interaction among variables, including an advanced diagnostic capabilities resulting in higher identification rates in developed countries, increased life expectancy broadens the population at risk in developed countries, and greater exposure to risks tied to accelerated urbanization and lifestyle shifts (28). A negative correlation was identified between PAH incidence and SDI regions. Regions with low SDI exhibited the highest ASIR, ASMR, while High SDI regions exhibiting the lowest ASIR, ASMR and ASDR. High SDI regions likely gained advantages from established healthcare infrastructure, successful public health efforts, and increased recognition of PAH risks and symptoms (29). For example, the high incidence rates in Sub-Saharan Africa and parts of Asia are probably due to a combination of genetic predisposition, HIV infection, congenital heart disease, and limitations within healthcare system (30). Central Asia experiences high mortality rates with an increasing ASM, primarily among patients with connective tissue diseases (particularly systemic sclerosis, SSc), portal hypertension, exposed to disease-causing drugs or toxins, and infected with *Schistosoma mansoni* (31, 32). Despite improvements in high SDI regions, the persistent disparity in mortality rates between low- and high-SDI areas highlights ongoing health inequities that must be addressed through targeted public health strategies and resource allocation. This underscores the intricate relationship between socio-economic factors and PAH outcomes, highlighting the impact of socio-demographic disparities on disease burden. Notably, across all SDI regions, there is an overall decline in both ASMR and ASDR.

These findings highlight the complex interplay of demographic, gender, and socioeconomic factors in shaping the global epidemiology of PAH, necessitating a multifaceted approach to disease prevention and management. However, the findings of this study ought to be interpreted with due consideration of the limitations of the study itself. Firstly, the validity of the estimates might be affected by the consistency and accessibility of data sources across diverse nations (33). In certain economically developing nations, the scarcity of accurate data on the epidemiological characteristics of PHA, combined with the insufficient cases, may result in a lower estimation of the actual global burden. Secondly, the GBD methodology is based on a number of presuppositions and computational models, which may result in a degree of inaccuracy within the produced estimates (34). Although the GBD database utilizes robust statistical techniques to mitigate these uncertainties, the findings are deemed to be the most accurate approximations currently available based on the existing evidence.

## Conclusion

In conclusion, the global burden of PAH persists as a significant public health concern, exhibiting notable disparities across regions, nations, and SDI categories. These findings highlight the imperative for the development of targeted preventative and therapeutic strategies that are tailored to the specific requirements of diverse, populations. Strengthening healthcare infrastructure, encouraging healthy behaviors, and addressing socioeconomic inequalities are addressed as a means of mitigating the global impact of PAH. Future research should prioritize optimize the identification and implementation of the most effective preventative and management for PAH, with a particular focus on regions with a high prevalence of the disease. Similarly, efforts should focus on unraveling the intricate relationships between genetic predispositions, Exposures to the environment, and Lifestyle related factors that contribute to the pathogenesis of PAH.

## Data Availability

The original contributions presented in the study are included in the article/Supplementary material, further inquiries can be directed to the corresponding authors.

## Footnotes

^1^ https://vizhub.healthdata.org/gbd-results/

^2^ https://ghdx.healthdata.org/gbd-2021

^3^ https://www.healthdata.org/gbd/methods-appendices-2021

## Supplementary material

SUPPLEMENTARY FIGURE 1 Results from clustering EAPC values for age-standardized death and DALY rates associated with PAH between 1990 and 2021. EAPC, estimated annual percentage change; DALYs, disability-adjusted life years; PAH, pulmonary arterial hypertension.

SUPPLEMENTARY FIGURE 2 The age-specific numbers and ASMRs of PAH by SDI regions in 1990 and 2021. **(A)** ASMR in 1990. **(B)** ASMR in 2021. ASMR, age-standardized death rate; PAH, pulmonary arterial hypertension; SDI, Social-Demographic Index.

SUPPLEMENTARY FIGURE 3 The age-specific numbers and ASDRs of PAH by SDI regions in 1990 and 2021. **(A)** ASDR in 1990. **(B)** ASDR in 2021. DALYs, disability-adjusted life years; ASDR, age-standardized DALYs rate; PAH, pulmonary arterial hypertension; SDI, Social-Demographic Index.
