# Supplementary figures and images for: Global, regional, and national trends in pulmonary arterial hypertension burden, 1990–2021: findings from the global burden of disease study 2021
Source: Front Public Health. 2025 May 29;13:1516365. doi: 10.3389/fpubh.2025.1516365 (PMC12158705; doi:10.3389/fpubh.2025.1516365)

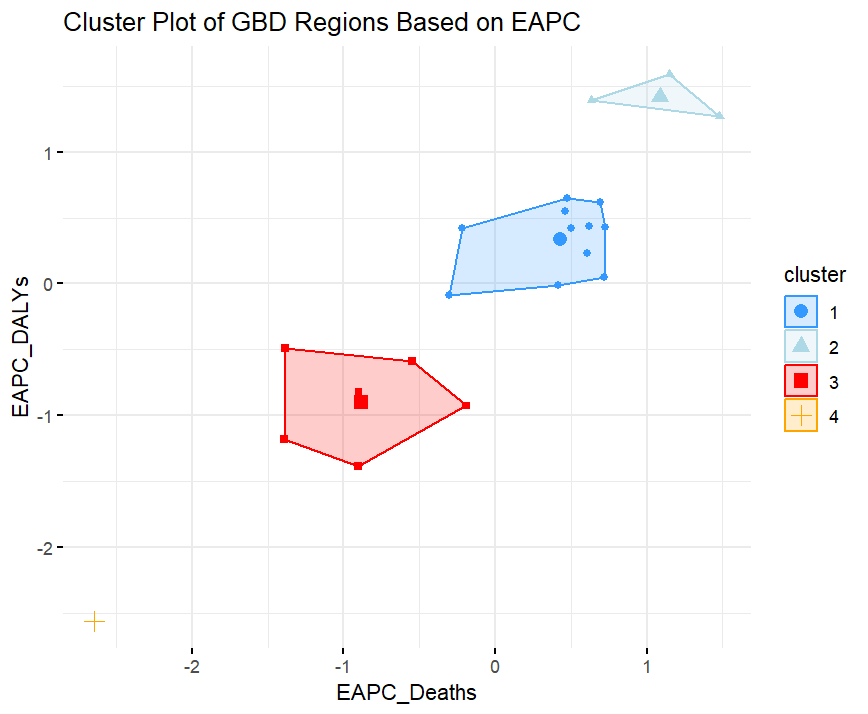

Supplement: SUPPLEMENTARY FIGURE 1 — Results from clustering EAPC values for age-standardized death and DALY rates associated with PAH between 1990 and 2021. EAPC, estimated annual percentage change; DALYs, disability-adjusted life years; PAH, pulmonary arterial hypertension. [file Image_1.tif]

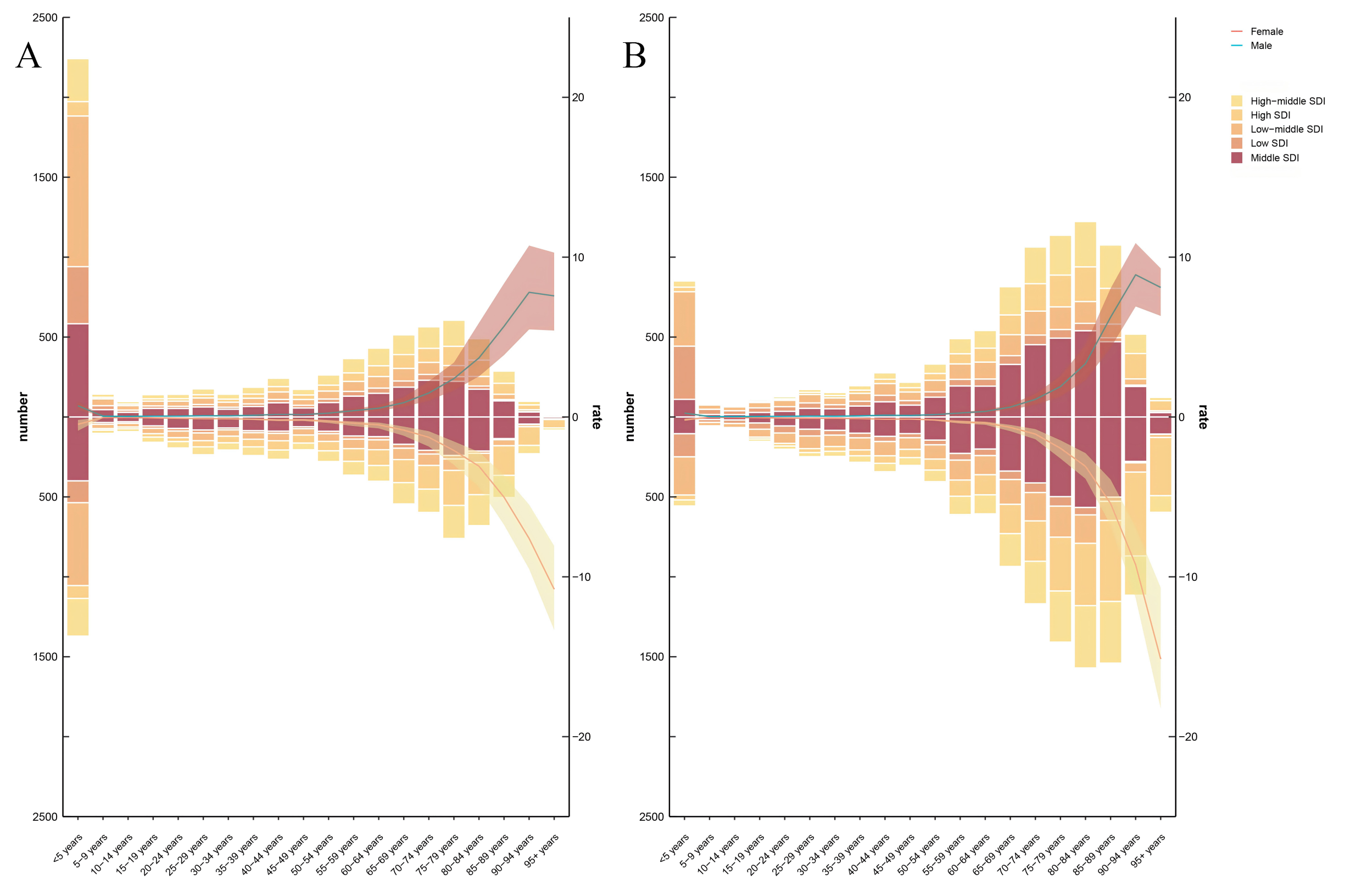

Supplement: SUPPLEMENTARY FIGURE 2 — The age-specific numbers and ASMRs of PAH by SDI regions in 1990 and 2021. (A) ASMR in 1990. (B) ASMR in 2021. ASMR, age-standardized death rate; PAH, pulmonary arterial hypertension; SDI, Social-Demographic Index. [file Image_2.tif]
